# Adipokines and the Right Ventricle: The MESA-RV Study

**DOI:** 10.1371/journal.pone.0136818

**Published:** 2015-09-08

**Authors:** Michael O. Harhay, Jorge R. Kizer, Michael H. Criqui, João A. C. Lima, Russell Tracy, David A. Bluemke, Steven M. Kawut

**Affiliations:** 1 Penn Cardiovascular Institute, Perelman School of Medicine, University of Pennsylvania, Philadelphia, Pennsylvania, United States of America; 2 Department of Medicine, Perelman School of Medicine, University of Pennsylvania, Philadelphia, Pennsylvania, United States of America; 3 Center for Clinical Epidemiology and Biostatistics, Perelman School of Medicine, University of Pennsylvania, Philadelphia, Pennsylvania, United States of America; 4 Department of Medicine, Department of Epidemiology and Population Health, Albert Einstein College of Medicine, Bronx, New York, United States of America; 5 Department of Family Medicine and Public Health, University of California San Diego, San Diego, California, United States of America; 6 Department of Medicine, Johns Hopkins University School of Medicine, Baltimore, Maryland, United States of America; 7 Department of Laboratory Medicine, University of Vermont School of Medicine, Burlington, Vermont, United States of America; 8 Radiology and Imaging Sciences, National Institute of Biomedical Imaging and Bioengineering, National Institutes of Health/Clinical Center, Bethesda, Maryland, United States of America; Indiana University, UNITED STATES

## Abstract

**Objective:**

Obesity is associated with changes in both right (RV) and left (LV) ventricular morphology, but the biological basis of this finding is not well established. We examined whether adipokine levels were associated with RV morphology and function in a population-based multiethnic sample free of clinical cardiovascular disease.

**Methods:**

We examined relationships of leptin, resistin, TNF-α, and adiponectin with RV morphology and function (from cardiac MRI) in participants (n = 1,267) free of clinical cardiovascular disease from the Multi-Ethnic Study of Atherosclerosis (MESA)-RV study. Multivariable regressions (linear, quantile [25^th^ and 75^th^] and generalized additive models [GAM]) were used to examine the independent association of each adipokine with RV mass, RV end-diastolic volume (RVEDV), RV end-systolic volume (RVESV), RV stroke volume (RVSV) and RV ejection fraction (RVEF).

**Results:**

Higher leptin levels were associated with significantly lower levels of RV mass, RVEDV, RVESV and stroke volume, but not RVEF, after adjustment for age, gender, race, height and weight. These associations were somewhat attenuated but still significant after adjustment for traditional risk factors and covariates, and were completely attenuated when correcting for the respective LV measures. There were no significant interactions of age, gender, or race/ethnicity on the relationship between the four adipokines and RV structure or function.

**Conclusions:**

Leptin levels are associated with favorable RV morphology in a multi-ethnic population free of cardiovascular disease, however these associations may be explained by a yet to be understood bi-ventricular process as this association was no longer present after adjustment for LV values. These findings complement the associations previously shown between adipokines and LV structure and function in both healthy and diseased patients. The mechanisms linking adipokines to healthy cardiovascular function require further investigation.

## Introduction

Obesity is an increasingly common public health problem. As of 2010, 35.5% of adults in the United States were obese [1], while 56.1% of adults met the metabolic syndrome criterion for an elevated waist circumference [2]. Obesity has been independently associated with left ventricular (LV) hypertrophy and dilation [3–5], and increasing body weight is associated with a 40 to 60% higher risk for incident heart failure [6, 7].

There are also significant implications of obesity in terms of right ventricular (RV) morphologic and functional changes, but these have only been recognized more recently. Prior work has shown that being overweight or obese is associated with increased RV mass and RV end-diastolic volume (RVEDV) and lower RV ejection fraction (RVEF) in the Multi-Ethnic Study of Atherosclerosis (MESA), independent of the respective LV parameter [8]. Others have shown similar findings in smaller cohorts [9, 10].

Adipokines are bioactive proteins produced by the adipose compartment that have wide-ranging effects across organs and tissues. Adiponectin and leptin are secreted primarily by adipocytes while the stromovascular tissue surrounding fat secretes tumor necrosis factor—alpha (TNF-α) and macrophages secrete resistin [11]. These adipokines have been proposed as links between adiposity and insulin resistance, glucose dysregulation, and cardiovascular disease [12]. Laboratory studies have shown that, apart from their direct influences on glucose and lipid metabolism, these adipose-derived molecules exert direct actions on vascular cells as well as cardiomyocytes. Recently, investigators showed that higher leptin was possibly associated with greater RV mass and larger RVEDV in healthy women [10, 13]. Additionally, a recent study in the MESA cohort found that higher levels of leptin are associated with more favorable values of several measures of LV structure and function [14]. Collectively, these findings suggest that adipokines are associated with the accumulation of triglycerides in nonadipose tissue compartments, such as the LV and potentially the RV [14, 15].

In this study we aimed to determine the associations between four adipokines (leptin, resistin, TNF-α, and adiponectin) with measures of RV structure and function measured by cardiac magnetic resonance imaging (MRI) among a large, nationally representative multi-ethnic adult population without clinical cardiovascular disease.

## Methods

### Subjects

The MESA is a multicenter prospective cohort study designed to investigate the prevalence, correlates, and progression of subclinical cardiovascular disease in Caucasians, African-Americans, Hispanics, and Chinese-Americans without clinical cardiovascular disease at baseline [16]. In 2000–2002, MESA recruited 6,814 men and women aged 45–84 years old from six communities in the United States: Forsyth County, North Carolina; Northern Manhattan and the Bronx, New York; Baltimore City and Baltimore County, Maryland; St. Paul, Minnesota; Chicago, Illinois; and Los Angeles, California. Exclusion criteria included clinical cardiovascular disease (physician diagnosis of heart attack, stroke, transient ischemic attack, heart failure, angina, current atrial fibrillation, any cardiovascular procedure), weight > 136 kilograms (kgs) (300 pounds), pregnancy, or impediment to long-term participation.

The study sample for the current study utilizes data from overlapping ancillary studies from the MESA. Specifically, of the 6,814 MESA participants, 5,098 had cardiac MRIs at baseline, of which 5,004 were interpretable for LV measures. The MESA-RV Study is an ancillary study that selected 4,634 of the interpretable LV MRI scans (without regard to age, sex, or race) for evaluation of RV structure and function. Of these, 4,424 had interpretation attempted and 4,204 were available and interpretable for RV morphology. The MESA Body Composition ancillary study analyzed stored venous blood for the adipokines adiponectin, leptin, resistin and TNF-α on a random sample of 1,964 participants from the baseline MESA cohort. Of these, 1,267 participants had interpretable RV MRI image data and complete covariate data, comprising the final sample for the current study.

The protocols of MESA and all studies described herein were approved by the Institutional Review Boards of collaborating institutions and the National Heart Lung and Blood Institute (NHLBI).

### Data Collection

Race and ethnicity was self-reported during the baseline MESA exam according to 2000 US Census criteria as race (Caucasian, African-American, Chinese) and ethnicity (Hispanic or non-Hispanic). Standard questionnaires were used to ascertain smoking status (classified as never, former, or current) and pack years. Height was measured to the nearest 0.1 cm with the subject in stocking feet. Weight was measured to the nearest 0.5 kg with the subject in light clothing using a balanced scale. Body mass index (BMI) was calculated [Weight (kg)/ Height (m)^2^]. Waist circumference was measured to the nearest 0.1 cm using a steel measuring tape (standard 4 ounce tension) from midway between the last rib and the iliac crest at normal breathing. Hip circumference was measured to the nearest 0.1 cm from the largest diameter of the hip. Resting blood pressure was measured three times using the Dinamap Monitor PRO 100 (Critikon, Tampa, FL) automated oscillometric device, and the average of the last two measurements was used. Hypertension was defined as systolic blood pressure ≥ 140 mmHg, diastolic blood pressure ≥ 90 mmHg or self-reported hypertension and current use of anti-hypertensive medication. Detailed methods for these measures are published elsewhere [17].

Spirometry measures were available for a subset of participants included in a MESA ancillary study. The MESA-Lung Study enrolled 3,965 of 4,484 eligible MESA participants who had consented to genetic analyses, undergone baseline measures of endothelial function, and attended an examination (with over-sampling of Chinese participants) in 2004 to 2006, of whom 1,063 were included for these analyses [18].

#### Cardiac Magnetic Resonance Imaging Measures

The cardiac MRI protocol and methods for interpretation of LV and RV parameters have been previously reported [19, 20]. All imaging was performed with 1.5-T magnets with a 4-element phased-array surface coil positioned anteriorly and posteriorly and ECG gating. Imaging consisted of fast gradient echo cine images with temporal resolution ≤ 50 milliseconds. Cardiac MRI examinations were transmitted to the reading center at Johns Hopkins University in Baltimore, MD, with the digital imaging and communications in medicine (DICOM) transfer protocol. Image analysis was done on Windows workstations with QMASS software (version 4.2; Medis, the Netherlands). Images were magnified to 250%. Image contrast was set to 55; image brightness was set to 55; and window width and level were set with the auto function in QMASS to minimum and maximum pixel values of 0 and 238, respectively.

The endocardial and epicardial borders of the RV were traced manually on the short-axis cine images at the end-systolic and end-diastolic phases. Full visualization of the correct placement of RV contours relied on evaluation of cine images to determine the demarcation between the right atrium and the RV. Contours were modified at basal slices of the heart by careful identification of the tricuspid valve so as to exclude the right atrium and to avoid overestimation of the volumes. The outflow tract was included in the RV volume.

RVEDV and RVESV were calculated using Simpson's rule by summation of areas on each slice multiplied by the sum of slice thickness and image gap. RV mass was determined at end-diastole as the difference between end-diastolic epicardial and endocardial volumes multiplied by the specific gravity of the heart (1.05 g/cm^3^) [20]. RVSV was calculated by subtracting RVESV from RVEDV; RVEF was calculated by dividing RVSV by RVEDV. The intrareader intraclass correlation coefficient from random, blinded rereads of 229 scans for RV mass was 0.94 and for RVEDV and RVEF from 230 scans was 0.99 and 0.89. The interreader intraclass correlation coefficients from random, blinded rereads of 240 scans for RV mass, RVEDV, and RVEF were 0.89, 0.96, and 0.80, respectively.

#### Laboratory methods

Total and HDL cholesterol, triglycerides, and glucose levels were measured from blood samples obtained after a 12-hour fast [21]. Diabetes was defined as fasting glucose ≥ 126 mg/dL or use of hypoglycemic medication. Fasting glucose between 100–125 mg/dL was considered impaired fasting glucose. Stored fasting blood samples were analyzed to provide levels of adiponectin, leptin, TNF-α and resistin. These adipokines were measured using Bio-Rad Luminex flow cytometry (Millepore, Billerica, Massachusetts) at the Laboratory for Clinical Biochemistry Research (University of Vermont, Burlington, Vermont). Average analytical coefficients of variation across several control samples for these analytes ranged from 6.0–13.0%.

### Statistical Analysis

In the descriptive tables, continuous variables are described as means and standard deviations and categorical variables are expressed as percentages. Each adipokine distribution was log transformed to reduce skew and the impact of outliers. Multivariable linear regression models were then used to assess the relationship of each of the adipokines with the five RV parameters: RV mass, RVEDV, RVESV, RVSV and RVEF. We examined three specifications of our initial model which included age, gender, race/ethnicity and either (1) height and weight, (2) BMI (continuous), or (3) BMI categories. The choice of body anthropometry used in the model did not affect the conclusions (i.e., the estimated regression coefficients). For the base model, adjustment for height and weight separately was chosen as it avoided the assumptions made in indexing the RV measures to certain parameters of body size (e.g., body surface area), while accounting for differences in body size between participants. Additional covariates were chosen based on (i) their independent p-values (less than 0.20 to avoid beta error), (ii) they impacted the estimated coefficient of the independent variable (i.e., the adipokine of interest by at least 10% indicating confounding) or (iii) because of their known biological roles with the RV. The fully adjusted model contained continuous measures of blood pressure, total cholesterol, HDL, triglycerides, waist and hip circumference, an indicator for smoking history (current, past, never), pack-years, diabetes mellitus, the use of hypertension medications, statin use and socioeconomic status (measured as highest level of educational attainment).

In the final model, adjustment for LV parameters was performed to (i) account for the contribution of LV abnormalities to RV changes (for example, increased LV mass causing pulmonary venous hypertension leading to increased RV mass), (ii) to better adjust for differences in body size, and (iii) to examine RV-specific associations (rather than more general associations with bi-ventricular morphology). These models when adjusted for the respective LV parameters facilitate a better understanding of whether the associations are right ventricle specific. To determine if the associations between the adipokines and RV measures were non-linear, we estimated unadjusted and adjusted generalized additive models (GAM). A subset of the study sample with available spirometry from MESA-Lung was also examined in further sensitivity analyses.

Statistical significance was defined as P < 0.05. As each adipokine analysis was considered an independent hypothesis, there was no correction made for multiple comparisons [22]. Analyses were performed using STATA 13.0 (StataCorp, College Station, Texas).

## Results

A total of 1,267 MESA participants participated in both the RV and adipokine ancillary studies, representing 18.9% of all MESA participants (Table 1). The mean age of the study sample was 61.3 ± 9.5 years and half were female. The mean BMI was 27.6 ± 4.7.kg/m^2^ and 40.5% were Caucasian, 26.0% were Hispanic, 19.0% were African American and 14.5% were Chinese American. Half were life-long non-smokers and 11.0% had diabetes mellitus, similar to those not included in this sub-study. The mean RV mass, RVEDV, RVESV, stroke volume and ejection fraction were 21.3 grams, 126.9 mL, 38.3 mL, 88.6 mL and 70.4%, respectively (Table 2). Pearson’s correlations between the RV and LV was the following: end-diastolic mass = 0.62, end-diastolic volume = 0.82, end-systolic volume = 0.64, stroke volume = 0.79, and ejection fraction = 0.47.

**Table 1:** Characteristics of the study sample. Participant characteristics are summarized as mean (SD) or n (%).

	Excluded	Study sample
	*n = 5*,*547*	*n = 1*,*267*
Age, years	62.34 (10.4)	61.32 (9.5)
Female	2966 (53.7%)	635 (49.3%)
Race/ethnicity		
Caucasian	2101 (38.0%)	521 (40.5%)
Chinese American	616 (11.1%)	187 (14.5%)
African American	1649 (29.8%)	244 (19.0%)
Hispanic	1161 (21.0%)	335 (26.0%)
Educational attainment		
No high school degree	1001 (18.2%)	224 (17.4%)
High school degree	1003 (18.2%)	233 (18.1%)
Some college, no bachelors	1580 (28.7%)	357 (27.8%)
Bachelors or graduate	1921 (34.9%)	472 (36.7%)
Height, cm	166.23 (10.0)	166.90 (9.9)
Weight, lbs	174.11 (39.0)	170.17 (34.6)
BMI, kg/m^2^	28.50 (5.6)	27.63 (4.7)
Systolic blood pressure, mmHg	126.79 (21.5)	125.74 (21.3)
Diastolic blood pressure, mmHg	71.74 (10.3)	72.67 (10.2)
Total cholesterol, mg/dL	193.93 (36.2)	195.12 (33.6)
HDL cholesterol, mg/dL	50.95 (14.8)	51.01 (15.0)
Triglycerides, mg/dL	131.10 (90.8)	133.70 (79.7)
Cigarette smoking status		
Never	2741 (49.8%)	677 (52.6%)
Former	2039 (37.0%)	448 (34.8%)
Current	726 (13.2%)	161 (12.5%)
Diabetes mellitus status		
Normal	4020 (73.0%)	972 (75.8%)
IGF	769 (14.0%)	170 (13.3%)
Untreated diabetes	141 (2.6%)	38 (3.0%)
Treated diabetes	577 (10.5%)	103 (8.0%)
Glucose, mg/dL	97.72 (31.2)	95.92 (26.0)
Smoking history, pack years	11.38 (21.1)	11.45 (26.8)
Resistin, pg/mL	17284.71 (11019.3)	15901.88 (6521.2)
Adiponectin, ng/mL	20290.98 (12752.3)	20944.11 (13378.1)
Leptin, pg/mL	23056.31 (22522.5)	19596.30 (22134.6)
TNF-α, pg/mL	6.51 (15.4)	5.61 (8.4)

**Table 2:** Left and right ventricle measures. LV = left ventricle, RV = right ventricle, SD = standard deviation.

	Mean	SD	Min	Max
LV end-diastolic mass, g	146.6	37.5	67.0	317.0
LV end-diastolic volume, mL	128.2	30.3	56.0	257.2
LV end-systolic volume, mL	40.4	16.2	10.1	144.1
LV stroke volume, mL	87.7	19.2	15.2	182.7
LV ejection fraction, %	69.1	7.3	18.9	87.3
RV end-diastolic mass, g	21.3	4.4	10.1	44.7
RV end-diastolic volume, mL	126.9	31.5	53.5	254.6
RV end-systolic volume, mL	38.3	14.9	4.9	100.1
RV stroke volume, mL	88.6	20.4	38.3	178.3
RV ejection fraction, %	70.4	6.5	47.1	91.3

To normalize the adipokine distributions in the multivariable linear regression models the adipokine values were natural log-transformed (Table 3). Higher leptin levels were associated with significantly lower levels of RV mass, RVEDV, RVESV and stroke volume after adjustment for age, gender, race, height and weight. Leptin levels were not associated with RVEF. These associations were somewhat attenuated but still significant after additional adjustment for education, blood pressure, cholesterol levels, triglycerides, current smoking status and smoking history (pack years), diabetes mellitus, waist & hip circumference, glucose levels, and indicator variables for statin use or hypertension medication. These observed associations between leptin and the RV were no longer significant after adjustment for the corresponding LV measures of function and morphology (Table 3). These results were not quantitatively different after adjustment for measures of lung-function derived from the spirometry exam (not shown). In addition, other covariates, including C-reactive protein, creatinine, estimated glomerular filtration rate and alcohol use, did not show evidence of confounding nor did they improve model fit. Examination of plots from GAM models did not show evidence of non-linear associations. There were no significant interactions between any of the adipokines with age, gender or race/ethnicity or between leptin and BMI.

**Table 3:** Adjusted linear regression coefficients and 95% confidence intervals of the association between adipokines and right ventricular structure and function. Model 1: Adjusted for age, gender, race, height and weight. Model 2: Model 1 + education, systolic and diastolic blood pressure, total & HDL cholesterol, triglycerides, current smoking status and pack years, diabetes, waist & hip circumference, glucose, and currently taking statins or hypertension medication. Model 3: Model 2 + respective LV measure. Abbreviations: RV = right ventricle, LV = left ventricle, LVEDV = left ventricular end-diastolic volume, LVESV = left ventricular end-systolic volume, LVEF = left ventricular ejection fraction, LVSV = left ventricular stroke volume.

	Log Leptin, pg/mL	Log Resistin, pg/mL	Log TNF-α, pg/mL	Log Adiponectin, ng/mL
	RV End-Diastolic Mass (g)			
Model 1	-0.34 (-0.56, -0.13) p = 0.002	0.03 (-0.42, 0.49) p = 0.88	0.06 (-0.26, 0.39) p = 0.70	0.06 (-0.28, 0.40) p = 0.73
Model 2	-0.30 (-0.51, -0.08) p = 0.008	0.02 (-0.44, 0.48) p = 0.95	0.11 (-0.22, 0.43) p = 0.52	-0.09 (-0.45, 0.26) p = 0.62
Model 2 + LV Mass	-0.13 (-0.34, 0.08) p = 0.21	0.05 (-0.38, 0.49) p = 0.81	0.01 (-0.30, 0.32) p = 0.94	-0.15 (-0.48, 0.19) p = 0.39
	RV End-Diastolic Volume (mL)			
Model 1	-4.14 (-5.58, -2.70) p < 0.001	-1.82 (-4.90, 1.26) p = 0.25	-0.80 (-3.00, 1.41) p = 0.48	1.81 (-0.49, 4.10) p = 0.12
Model 2	-3.81 (-5.25, -2.36) p < 0.001	-1.77 (-4.84, 1.31) p = 0.26	-0.53 (-2.71, 1.64) p = 0.63	0.90 (-1.49, 3.28) p = 0.46
Model 2 + LVEDV	-0.75 (-1.81, 0.30) p = 0.16	-0.49 (-2.68, 1.71) p = 0.66	-0.30 (-1.85, 1.25) p = 0.71	-0.30 (-2.00, 1.39) p = 0.73
	RV End-Systolic Volume (mL)			
Model 1	-1.52 (-2.26, -0.78) p < 0.001	-0.78 (-2.35, 0.80) p = 0.33	0.35 (-0.78, 1.48) p = 0.54	-0.03 (-1.20, 1.15) p = 0.96
Model 2	-1.44 (-2.20, -0.69) p < 0.001	-0.84 (-2.43, 0.76) p = 0.31	0.42 (-0.71, 1.54) p = 0.47	-0.21 (-1.44, 1.03) p = 0.74
Model 2 + LVESV	-0.66 (-1.33, 0.02) p = 0.05	-0.93 (-2.34, 0.48) p = 0.20	0.11 (-0.89, 1.11) p = 0.83	-0.83 (-1.93, 0.26) p = 0.14
	RV Stroke Volume (mL)			
Model 1	-2.62 (-3.68, -1.55) p < 0.001	-1.04 (-3.31, 1.23) p = 0.37	-1.15 (-2.77, 0.48) p = 0.17	1.84 (0.15, 3.53) p = 0.03
Model 2	-2.36 (-3.43, -1.30) p < 0.001	-0.93 (-3.19, 1.33) p = 0.42	-0.95 (-2.54, 0.65) p = 0.25	1.10 (-0.65, 2.85) p = 0.22
Model 2 + LVSV	-0.51 (-1.26, 0.25) p = 0.19	0.59 (-1.00, 2.17) p = 0.46	-0.17 (-1.29, 0.95) p = 0.77	0.91 (-0.31, 2.14) p = 0.14
	RV Ejection Fraction (%)			
Model 1	0.05 (-0.36, 0.46) p = 0.82	0.22 (-0.65, 1.08) p = 0.63	-0.37 (-0.99, 0.25) p = 0.24	0.43 (-0.22, 1.07) p = 0.19
Model 2	0.06 (-0.36, 0.48) p = 0.78	0.27 (-0.61, 1.15) p = 0.55	-0.35 (-0.97, 0.27) p = 0.27	0.38 (-0.30, 1.06) p = 0.27
Model 2+ LVEF	-0.03 (-0.42, 0.35) p = 0.86	0.52 (-0.29, 1.33) p = 0.21	-0.11 (-0.68, 0.47) p = 0.72	0.63 (0.00, 1.25) p = 0.05

Regression analyses showed that there were no significant associations between adiponectin, resistin and TNF-α and RV measures with one exception. Adiponectin showed a positive association with RVEF in the LV adjusted models, but not without accounting for LVEF. GAM plots did not reveal significant non-linearity in the associations of any of the adipokines with RV parameters.

## Discussion

This is the first study to examine the association between adipokines and the RV, contributing to the growing literature supporting the relationship of adipokines with cardiovascular function and disease [11, 15, 23]. In this cross-sectional study of a large cohort of individuals from four distinct ethnic groups from across the United States, higher levels of leptin were associated with significantly lower RV mass, RVEDV, RVESV, and RVSV independent of anthropometric measures and other confounders, but not after accounting for LV function. Conversely, there were no persistent associations between these RV measures and adiponectin, resistin or TNF-α, which also align with recent findings in the LV in the MESA cohort [14].

Plasma leptin is produced by adipose tissue and regulates body fat stores and food intake. Leptin has pleitropic actions in the central nervous system and studies have shown both hypertrophic and cardioprotective actions on the LV myocardium. Some animal models have shown that leptin increases LV hypertrophy [24] whereas others showed that leptin inhibits the hypertrophic response [25, 26]. Human studies have also reported conflicting roles of leptin on cardiac myocyte mass. While some studies have shown a positive relationship between plasma leptin levels and LV mass [10, 13, 27], other studies suggest an inverse relationship as shown in our study in the RV. Lieb et al. found that higher leptin levels were associated with lower LV mass in community-based older adults [28, 29]. Similar findings were demonstrated in a population-based sample of 410 adults from Spain [30] and in overweight sedentary postmenopausal women [31]. These data correspond with other findings in obese adults (showing increased ventricular mass), which is characterized by leptin resistance [28, 29] and correspond with the obesity-related increases in RV mass shown in the MESA cohort [8, 32].

It appeared that leptin had a similar relationship with both LV and RV mass, since the association was not present after additional adjustment for LV mass. It is also possible that leptin affected the RV through changes in LV mass and left-ventricular end-diastolic pressure. The MESA cohort includes individuals free of clinical cardiovascular disease, which might explain the inverse associations with RV mass, whereas studies showing adverse effects of leptin are from patients with clinical cardiovascular disease. Adiponectin conversely showed a positive association with RVEF but not RV mass in the LV adjusted models. Adiponectin has been shown to exert vasculoprotective, anti-atherosclerotic, and anti-thrombotic effects through modulation of endothelial cells, smooth muscle cells, macrophages, and platelets [33]. Adiponectin has antihypertrophic effects and protects against ischemia-reperfusion injury in laboratory models [23]. Likewise, reduced plasma levels of adiponectin have been reported in diabetics and patients with coronary artery disease and increased carotid intima-media thickness. Taken together, the cardiovascular findings with adiponectin appear to be complex and not very well understood, showing an inverse relationship in healthy younger cohorts but a positive association with older cohorts or cohorts with prevalent disease [34–37].

There are several limitations to our study. Foremost, cross-sectional studies are unable to establish temporality, and thus casual inferences cannot be made. Additionally, not all MESA participants were able to tolerate cardiac MRI and some readings were uninterpretable. However, there were only slight differences between those individuals included in our study sample and those not (Table 1). A strength of this study is that we are uniquely able to examine adults over a spectrum of ages and of various races and ethnicities, increasing generalizability. In participants without clinical cardiovascular disease, cardiac MRI may be more sensitive for detection of subtle cardiovascular changes than other methods, such as echocardiography or right heart catheterization. To our knowledge, there is no other large population-based multi-ethnic cohort that has completed MRI-based RV measures.

In conclusion, the associations between RV structure and function in clinical cardiovascular disease-free individuals observed in this study complement the associations shown between adipokines and LV structure and function in diseased and healthy patients [14]. Yet, the understanding of the mechanisms linking adipokines to healthy vascular function is still inchoate and has to date largely been focused on leptin. This analysis adds to the growing literature on these mediators released from adipose tissue and how they modulate cardiac and vascular processes in cardiovascular disease free individuals.
